# Interferon-****γ**** Triggers Hepatic Stellate Cell-Mediated Immune Regulation through MEK/ERK Signaling Pathway

**DOI:** 10.1155/2013/389807

**Published:** 2013-05-07

**Authors:** Xiaodong Gu, Yan Wang, Jianbin Xiang, Zongyou Chen, Lianfu Wang, Lina Lu, Shiguang Qian

**Affiliations:** ^1^Department of General Surgery, Huashan Hospital, Fudan University, Shanghai 200040, China; ^2^Department of General Surgery, Cleveland Clinic Foundation, Cleveland, OH 44195, USA; ^3^Department of Immunology, Lerner Research Institute, Cleveland Clinic Foundation, Cleveland, OH 44195, USA

## Abstract

Hepatic stellate cells (HSCs) interact with immune cells to actively participate in regulating immune response in the liver which is mediated by the effector molecules, including B7-H1. We demonstrated here that expression of B7-H1 on HSCs was markedly enhanced by interferon-(IFN-) **γ** stimulation. IFN-**γ** stimulated HSCs inhibited T-cell proliferation via induction of T-cell apoptosis (22.1% ± 1.6%). This immunosuppressive effect was inhibited by preincubation with an anti-B7-H1 antibody, or inhibitor of the MEK/ERK pathway inhibited IFN-**γ** mediated expression of B7-H1. Thus, regulation of B7-H1 expression on HSCs by IFN-**γ** represents an important mechanism that regulates immune responses in the liver favoring tolerogenicity rather than immunogenicity. Involvement of MEK/ERK pathway provides a novel target for therapeutic approaches.

## 1. Introduction

Hepatic tolerance was initially suggested by spontaneous acceptance of liver transplants a cross major MHC disparity without the requirement for immunosuppressive therapy in many species, as well as by induction of tolerance to antigens delivered by way of portal vein or oral route [1–3]. In addition, the spontaneous acceptance of liver allograft can suppress the rejection of subsequent other organ grafts such as heart or kidney from the same donor [4, 5]. Interestingly, although liver allografts are accepted, hepatocytes transplants are promptly rejected, suggesting a crucial role of liver nonparenchymal cells in protecting parenchymal cells from immune attacks. We have demonstrated that hepatic stellate cells (HSCs), abundant liver tissue stromal cells known for participating in liver fibrogenesis, can protect the cotransplanted islets allografts from rejection [6]. However, quiescent and B7-H1 gene knockout (KO) HSCs lost the protective effect on co-transplanted islet allografts, indicating a critical role of B7-H1 in immune regulatory activity of HSCs, which may represent one of the mechanisms that regulate immune responses in the liver favoring tolerance rather immunogenicity [7]. However, little is known about the regulatory mechanisms of B7-H1 expression in HSCs.

Interferon-(IFN-) *γ* is a proinflammatory cytokine that is, mainly produced by T cells and natural killer (NK) cells and has been shown to participate in regulation of antiviral and antitumor immunity [8]. Thus, in cancer microenvironment, a cellular process involving the release of inflammatory mediators including IFN-*γ* [9] and subsequently induction of various cellular proteins such as B7-H1 in cancer cells was reported. Overwhelming data indicate that cancer-associated B7-H1 in murine cancer model facilitated apoptosis of cancer-reactive T cells [10]. B7-H1 expression was enhanced on malignant plasma cells from multiple myeloma patients by IFN-*γ* and Toll-like receptor stimulation via MEK/ERK-dependent and MyD88/TRAF6-dependent pathways and can inhibit T-cell responses [11]. JAK/STAT pathway was also involved in induction of B7-H1 expression in response to IFN-*γ* in a human lung cancer cell line [12]. However, it remains unclear whether molecular mechanisms are involved in IFN-*γ*-induced B7-H1 expression in HSCs.

In this study, we demonstrated that HSCs expressed B7-H1 in response to IFN-*γ* stimulation in a dose- and time-dependent manner at transcriptional level, and the MEK/ERK pathway is responsible for the IFN-*γ*-induced expression of B7-H1 in HSCs. And stimulation of HSCs with IFN-*γ* reduced T-cell proliferation and promoted T-cell apoptosis.

## 2. Materials and Methods

### 2.1. Mice and Reagents

C57BL/6 (B6; H-2^b^) and BALB/c (H-2^d^) mice were purchased from Shanghai Laboratory Animal Center of Chinese Academy of Sciences (Shanghai, China). Stat1 KO (129S6/SvEvTac-Stat^tm1Rds^) mice were obtained from Taconic (Germantown, NY, USA). IFN-*γ* R1 KO (B6.129S7-Ifngr1^tm1Agt^/J) mice were purchased from the Jackson Laboratory (Bar Harbor, ME, USA). B7-H1 KO mice were kindly provided by Dr. Lieping Chen (Johns Hopkins University Medical School, Baltimore, MD, USA). Animals were fed with standard chow *ad libitum *and were used at 7–9 weeks of age. The animal experimental protocols were in accordance with Chinese Administration Rule of Laboratory Animal. Recombinant IFN-*γ*, cycloheximide (CHX), actinomycin D (ActD), phorbol myristate acetate (PMA), and U0126 were obtained from Sigma-Aldrich (St. Louis, MO, USA). SP600125 and LY294002 were purchased from Cell Signaling Technology (Danvers, MA, USA).

### 2.2. Preparation of HSCs

HSCs were isolated from the mice liver nonparenchymal cells as previously described [7]. The liver was perfused through the portal vein with collagenase IV (Life Technologies, Grand Island, NY, USA). The smashed cells were filtered through a nylon mesh. Subsequently, the HSCs were purified by Percoll density gradient centrifugation (Sigma-Aldrich) and cultured in complete medium supplemented with 20% fetal bovine serum for 7 to 14 days unless otherwise indicated. The purity of HSCs ranged from 90% to 95% measured by desmin immunostaining and the typical light microscopic appearance of the lipid droplets.

### 2.3. Flow Cytometric Analysis

Expression of cell surface molecules was detected on FACScan (BD Biosciences, San Jose, CA, USA) and analyzed using CellQuest software (BD Biosciences). Cells were stained with the following monoclonal antibodies (mAbs): FITC-B7-H1 (eBioscience, San Diego, CA, USA) and FITC-CD3 (BD Biosciences). Isotype-matched irrelevant mAbs were used as negative controls. Apoptosis was assessed by PE-Annexin V staining (BD Biosciences).

### 2.4. Reverse Transcription Polymerase Chain Reaction (RT-PCR)

Total RNA was extracted from HSCs with TRIzol reagent (Life Technologies) according to the manufacturer's instructions. RNA was then reverse-transcribed into cDNA, using random primers and SuperScript II reverse transcriptase (Life Technologies). For semiquantitative RT-PCR, the PCR amplification was performed using Taq DNA polymerase (Life Technologies). PCR products were analyzed on agarose gels stained with ethidium bromide and photographed. B7-H1 cDNA was amplified by RT-PCR using the forward primer 5′-CTGTAGAACGGGAGCTGGAC-3′ and the reverse primer 5′-TGGACTTTCAGCGTGATTCG-3′.

### 2.5. Western Blot Analyses

HSCs were suspended in lysis buffer (20 mM Tris-HCl, pH 7.8, 50 mM NaCl, 5 mM EGTA, and 1% v/v Triton X-100) containing freshly added protease and phosphatase inhibitors (1 mM phenylmethylsulfonyl fluoride, 1 *μ*M leupeptin, 2 *μ*M aprotinin, 1 mM sodium orthovanadate, and 20 mM glycerophosphate). Lysates were clarified by centrifugation at 4°C and protein concentration determined by Bio-Rad protein assay (Hercules, CA, USA). Equal quantities of proteins were separated by SDS-PAGE, transferred to PVDF membrane, and sequentially incubated with primary antibodies and HRP-conjugated secondary antibodies, followed by enhanced chemiluminescence detection. Anti-phospho-ERK1/2 (Thr202/Tyr204) and anti-ERK2 antibodies were purchased from Cell Signaling Technology.

### 2.6. Mixed Lymphocyte Reaction (MLR)

Nylon wool-eluted spleen T cells (2 × 10^5^) from BALB/c mice were used as responders. *γ*-irradiated (20 Gy) DCs derived from B6 bone marrow were used as stimulators. Cultures were maintained in complete medium for 3 days at 37°C in 5% humidified CO_2_. [^3^H]-thymidine (0.5 *μ*Ci/well) was added for the final 18 hours of culture. Cells were harvested onto glass fiber disks using an automated system, and incorporation of [^3^H]-thymidine into DNA was assessed by Wallac 1450 liquid scintillation counter (PerkinElmer, Boston, MA, USA). Results were expressed as mean counts per minute (cpm) ± 1 SD. To examine the effect of HSCs on T-cell proliferation, *γ*-irradiated (50 Gy) HSCs were added into cultures at the beginning of the culture.

### 2.7. Statistical Analysis

Statistical analysis was performed with Stata 8.0 software (Stata, College Station, TX, USA). The data was given as mean ± 1 SD. Statistical comparisons between groups were performed using a one-way ANOVA followed by a Scheff's test, as appropriate. Values of *P* < 0.05 were considered statistically significant.

## 3. Results

### 3.1. IFN-*γ* Induces B7-H1 Expression in HSCs

**Figure 1:**
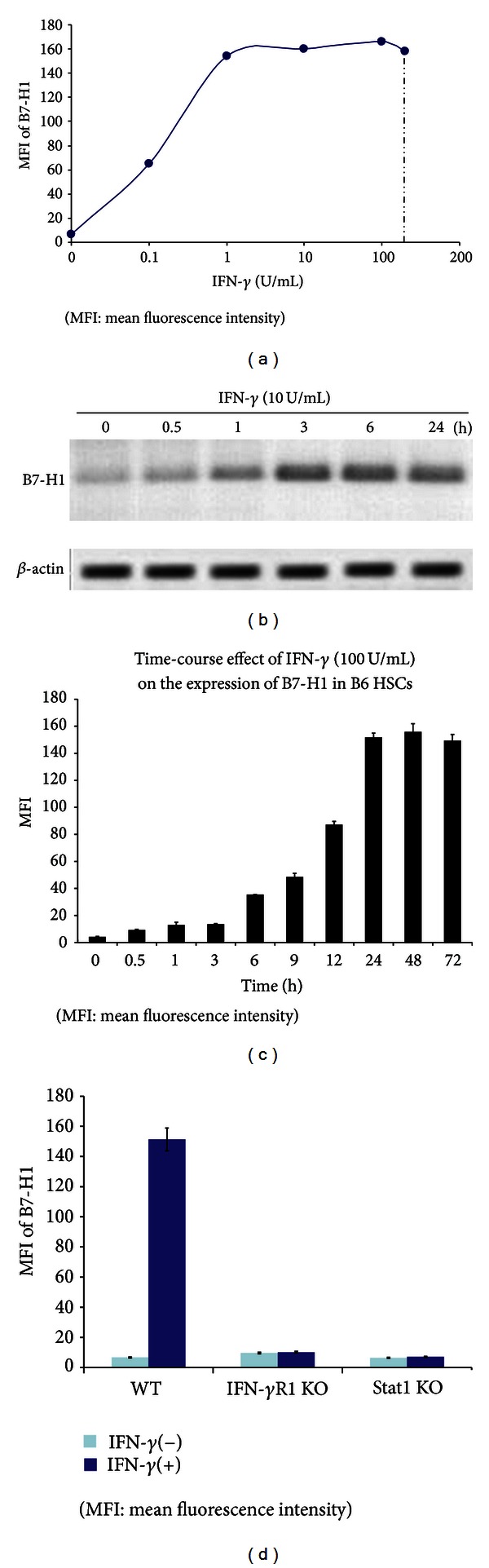
Expression of B7-H1 on HSCs in response to INF-*γ* stimulation. (a) HSCs isolated from B6 mice were exposed to graded concentrations of IFN-*γ* (0.1–200 U/mL) for 24 hours in vitro and stained with anti-B7-H1 mAb and analyzed by flow cytometry. (b) HSCs were treated with IFN-*γ* (10 U/mL) for varying times (0.5–24 hours) and analyzed by RT-PCR. (c) HSCs were incubated with IFN-*γ* (100 U/mL) for the indicated times, and the expression patterns were analyzed by flow cytometry. (d) HSCs isolated from wild type (WT) or IFN-*γ*R1 KO mice or Stat1 KO mice (all on B6 background) were exposed to IFN-*γ* (100 U/mL) for 48 hours. Cells were stained using anti-B7-H1 mAb and analyzed by flow cytometry. The data are representative of two separate experiments.

Quiescent HSCs isolated from B6 mice expressed very low B7-H1. However, expression of B7-H1 was markedly upregulated following exposure to IFN-*γ*. To determine the dose- and time-dependent effects of IFN-*γ*-induced B7-H1 expression, we treated HSCs with various concentrations of IFN-*γ* (0.1–200 U/mL) for 24 hours or at the same concentration but various duration. The result showed that increase in expression of B7-H1 was correlated with the IFN-*γ* concentration (Figure 1(a)). As shown in Figures 1(b) and 1(c), B7-H1 expression initiated to be increased following exposure to IFN-*γ* for as short as 0.5 hours and reached at the maximum after stimulation for 24–48 hours. 

IFN-*γ* receptor (R) contains IFN-*γ*R1 binding chain and internal IFN-*γ*R2 transducing chain [13]. Expression of B7-H1 on HSCs that were isolated from IFN-*γ*R1 KO mice showed no response to IFN-*γ* (Figure 1(d)), indicating that B7-H1 is a product of the IFN-*γ* signaling. This was supported by the fact that B7-H1 expression in response to IFN-*γ* stimulation was almost entirely impeded on HSCs isolated from Stat1 KO mice (Figure 1(d)), since Stat1 is a key transcription mediator for IFN-*γ* signaling [8].

### 3.2. Involvement of MEK/ERK Pathway in IFN-*γ* Induced B7-H1 Expression

**Figure 2:**
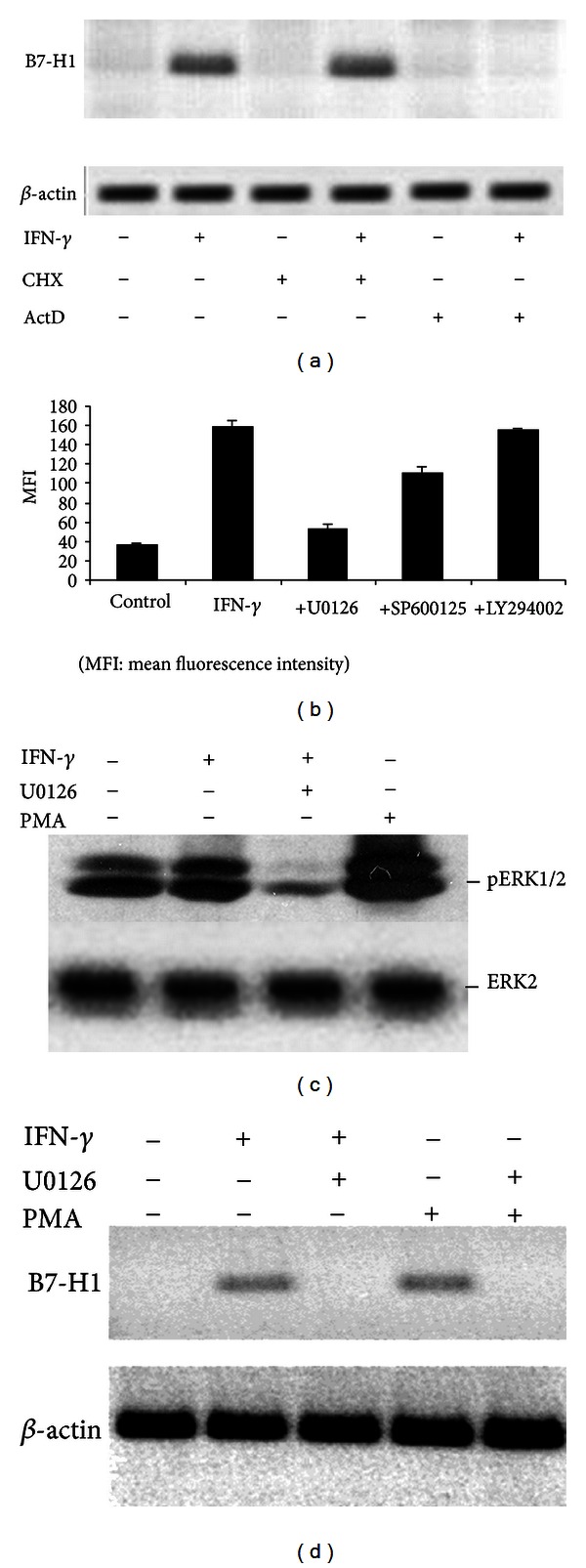
MEK/ERK-dependent B7-H1 expression in HSCs. (a) B7-H1 mRNA levels in HSCs measured by RT-PCR after exposure to IFN-*γ* (10 U/mL) for 6 hours with or without 10 *μ*g/mL CHX or 10 *μ*g/mL ActD for 90 minutes. (b) Flow cytometric analysis of B7-H1 expression in control HSCs (without IFN-*γ* stimulation) and after 24 hours incubation with IFN-*γ* with or without 1 hour pretreatment with signal transduction inhibitors, that is, 100 *μ*M U0126 (MEK1/2), 100 *μ*M SP600125 (JNK), and 100 *μ*M LY294002 (PI3 K). (c) Western blot analysis of ERK1/2 phosphorylation in HSCs after incubation with IFN-*γ* (10 U/mL) or 1 ng/mL PMA, with or without 1 hour preincubation with 100 *μ*M U0126. (d) RT-PCR analysis of B7-H1 mRNA levels in HSCs exposed for 24 hours to IFN-*γ* (10 U/mL) or 1 ng/mL PMA with or without 1 hour pre-incubation with 100 *μ*M U0126.

To understand which signaling pathway is involved in IFN-*γ*-induced B7-H1 expression in HSCs, we first assessed if RNA synthesis was required in this process by blocking RNA synthesis with ActD. Addition of ActD completely blocked B7-H1 mRNA synthesis in HSCs (Figure 2(a)). However, blocking protein synthesis with CHX had no effect on B7-H1 mRNA synthesis, indicating that de novo protein synthesis is not required for B7-H1 transcription. 

We then tried to determine the involved signaling pathways using several transduction pathway inhibitors (Figure 2(b)). Blocking of PI3 K with LY294002 did not reduce B7-H1 expression. However, blocking MEK1/2 with U0126 dramatically downregulated IFN-*γ*-induced B7-H1 expression. A slight reduction was also observed after blocking JNK with SP600125. 

To confirm that IFN-*γ* induced B7-H1 expression through MEK/ERK pathway in HSCs, we analyzed the phosphorylation of ERK1/2, showing that IFN-*γ* induced phosphorylation of ERK1/2, which was almost completely blocked by U0126 (Figure 2(c)). Phosphorylation of ERK1/2 was also identified in HSCs without exposure to IFN-*γ* (Figure 2(c)). These HSCs were activated with culture in vitro for 7 to 14 days. They might produce other factors that participated in phosphorylation of ERK1/2. Incubation of HSCs in PMA, a known activator of the MEK/ERK pathway, induced B7-H1 expression and ERK1/2 phosphorylation which were also blocked by U0126 (Figures 2(c) and 2(d)).

### 3.3. T-Cell Inhibition by IFN-*γ* Stimulated HSCs

**Figure 3:**
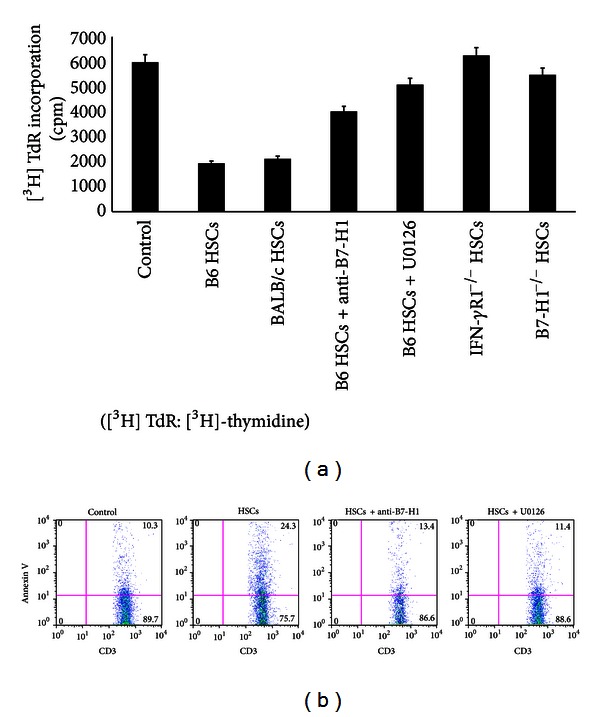
T-cell inhibition by HSCs. (a) B6, BALB/c, IFN-*γ*R1 KO, or B7-H1 KO HSCs cultured in uncoated plastics for 7 days were *γ*-irradiated (50 Gy) and added at the beginning of an MLR culture in which splenic T cells (2 × 10^5^) from BALB/c mice, and irradiated (20 Gy) B6 DCs were cultured at a final T-cell/DC/HSC ratio of 20 : 2 : 1 for 3 days. In some groups, irradiated HSCs pre-incubated with 100 *μ*M U0126 or anti-B7-H1. Controls were without HSCs. The data are representative of three separate experiments. (b) Cells following cultures for 2 or 3 days were double stained with FITC-anti-CD3 and PE-anti-Annexin V for flow analysis. The data demonstrated Annexin V expression in CD3+ populations. In some groups, irradiated HSCs pre-incubated with 100 *μ*M U0126 or anti-B7-H1. Controls were without HSCs. The data are representative of three separate experiments.

To test the ability of HSCs to suppress T-cell responses, HSCs were added into a MLR culture in which BALB/c splenic T cells were stimulated by B6 DCs. HSCs from either B6 or BALB/c mouse livers markedly suppressed thymidine uptake by T cells in a dose dependent fashion (Figure 3(a)), suggesting that the inhibitory effect of HSCs on T-cell response is not MHC specific. The inhibitory effect of HSCs on T-cell response was partially reversed when HSCs were pre-incubated with anti-B7-H1 antibody or with U0126 before being added to the culture (Figure 3(a)). HSCs from IFN-*γ*R1 KO mice and B7-H1 KO mice also lost the capacity to inhibit T-cell response (Figure 3(a)).

We speculated that HSCs induced T-cell hyporesponsiveness may result from apoptotic death of activated T cells. To address this, BALB/c splenic T cells were cultured for 3 days with irradiated allogeneic (B6) DCs in the presence or absence of activated B6 HSCs. Flow cytometric analysis of the cells that were double stained with anti-CD3 mAb and anti- Annexin V mAb confirmed that the activated HSCs enhanced incidence of T-cell apoptosis (Figure 3(b)). Apoptotic T cells markedly decreased when U0126 or anti-B7-H1 antibody was added during pre-incubation (Figure 3(b)). Thus, stimulation of HSCs with IFN-*γ* reduced T-cell proliferation and promoted T-cell apoptosis, via a MEK/ERK/B7-H1 pathway.

## 4. Discussion 

IFN-*γ* is an important proinflammatory cytokine mainly produced by T helper 1 cells and NK cells, mediating both innate and adaptive immune responses [14]. Recent accumulating evidence suggests that IFN-*γ* is also critical for tolerance induction in transplantation [13, 15, 16]. Liver allografts transplanted into wild type (WT) mice achieve long-term survival, whereas no WT allografts survived beyond 14 days in IFN-*γ* KO recipients or IFN-*γ*R KO allografts in WT recipients [13]. The underlying mechanisms are not completely understood. IFN-*γ* is an important modulator of cytotoxic T cells, macrophages, and NK cells, as well as the expression of MHC molecules. Many genes, including those for various chemokines, adhesion molecules, and costimulatory molecules, are transcriptionally activated in IFN-*γ* treated cells. Among these molecules, B7-H1 is broadly expressed on most lymphocyte lineage cells, normal tissue, and a variety of tumor cells by stimulated cytokines [17].

The role of B7-H1 as a coinhibitory ligand is consistent with its ability to interact with its receptor PD-1, which also binds to B7-DC on DCs [18]. There were conflicting data that have been reported on the role of B7-H1. Many studies in mouse islet, corneal, skin, and cardiac transplant models have demonstrated that the PD-1/B7-H1 pathway is required for the induction and maintenance of established graft tolerance [19]. However, in some in vivo settings, B7-H1 can costimulate T-cell responses [20]. We found that HSCs deficient in either B7-H1 or IFN-*γ*R1 largely lost the capacity to inhibit T-cell response, indicating that the immune regulation of HSCs requires IFN-*γ* stimulation, and that its downstream product, B7-H1, is a crucial effector molecule.

The present study revealed that exposure of HSCs to IFN-*γ* resulted in a dramatic increase in B7-H1 expression in dose- and time-dependent manners. We tried to inhibit several components of the pathways known to mediate IFN-*γ* signaling. Inhibition of MEK1/2 almost completely blocked IFN-*γ*-induced B7-H1 expression by HSCs. A partial inhibition of B7-H1 expression was observed when JNK was blocked with SP600125, but no effect could be observed when the PI3k pathway was blocked. U0126 was also able to block the inhibitory effect of IFN-*γ*-stimulated HSCs on T cells. Thus, the MEK/ERK pathway seems to be a major contributor responsible for IFN-*γ* induced expression of B7-H1 in HSCs. Several studies have previously established that, in addition to the classical JAK/Stat pathway, IFN-*γ* also activates MAPK [8]. p38 MAPK can activate Stat1 through phosphorylation of serine 727 [21]. ERK activates C/EBP dependent gene transcription through IFN-*γ* [22]. Lee et al. [23] demonstrated that MAPK and PI3 K pathways were involved in induction of B7-H1 expression in response to IFN-*γ* in dermal fibroblasts. Liu et al. [11] reported that MEK/ERK and MyD88/TRAF6 pathways were important for inducing B7-H1 expression in multiple myeloma plasma cells by IFN-*γ* and Toll-like receptor stimulation. Our data did not support a role for PI3 K pathway in induction of B7-H1 expression in HSCs, but we found the LPS did not induce B7-H1 expression in HSCs (data not shown). It is likely that IFN-*γ* signaling is mediated through different pathways depending on the cell types involved.

In summary, we have shown that B7-H1 is expressed on HSCs, is involved in inhibition of T-cell responses by these HSCs, and is upregulated by IFN-*γ* through the MEK/ERK pathway. These findings may provide new insights into better understanding of the mechanisms regarding how HSCs participate in hepatic tolerogenicity and help to develop novel strategies for induction of transplantation tolerance.
